# A randomized controlled trial for the effect of Modified Shenling Baizhu Powder on delaying the illness progress of COPD stable phase patients (GOLD 1–2 stages): A study protocol

**DOI:** 10.1097/MD.0000000000022700

**Published:** 2020-10-23

**Authors:** Yufei Liu, Xiaohong Xie, Wujun Wang, Keni Zhao, Wei Xiao, Jing Xiao, Jianqin Chen, Jurong Zeng, Keling Chen

**Affiliations:** Department of Respiratory Medicine, Hospital of Chengdu University of Traditional Chinese Medicine, Chengdu, China.

**Keywords:** Chinese herbal medicine, chronic obstructive pulmonary disease, Modified Shenling Baizhu Powder, randomized controlled trial

## Abstract

**Introduction:**

As one of the most prominent public health and medical problems, Chronic Obstructive Pulmonary Disease (COPD) has a serious impact on the quality of life of participants and may even be life-threatening. While modern medicine has worked well to alleviate the symptoms of COPD, the current situation with this chronic disease is not encouraging. Lung-spleen qi deficiency syndrome is one of the common forms of COPD and the traditional Chinese medicine formula Modified Shenling Baizhu Powder is very frequently used in the treatment of this syndrome. However, no direct evidence is available to support the efficacy and safety of Modified Shenling Baizhu Powder for COPD treatment.

**Methods:**

The study is a prospective, randomized, placebo-controlled, double-blind trial in which 270 eligible participants will be randomly assigned to either the experimental or control group in a 1:1 ratio. Both groups will receive the standard Western medication. Meanwhile, participants in the experimental group will undergo Modified Shenling Baizhu Powder, while those in the control group will undergo a matched placebo. The course of treatment is 6 months with 12 months of follow-up. Primary outcome is the forced expiratory volume in 1 second (FEV1) after bronchodi-lator use. The secondary outcomes include the declines and the between-group difference in the change from baseline to 18 months in FEV1 before bronchodilator use; the forced vital capacity (FVC), FEV1/FVC, FEV1%pred after bronchodilator use, modified British medical research council, COPD Assessment Test, St George's Respiratory Questionnaire (SGRQ); frequency, interval, duration and severity of COPD exacerbations; time to first COPD exacerbation; administration of rescue medication and a cost-effectiveness analysis; Smoking status. A safety assessment will also be performed during the trial.

**Discussion:**

The results of this trial will provide comprehensive evidence of the efficacy of Modified Shenling Baizhu Powder for early-stage COPD and the potential mechanism by which Modified Shenling Baizhu Powder acts, which may provide reference for the treatment plan of COPD participants.

**Trial registration:**

ChiCTR2000037873, Registered 2 September 2020

## 1 Introduction

Chronic obstructive pulmonary disease (COPD) is the third leading cause of death worldwide.^[1]^ and contributes to high morbidity and mortality in China.^[2]^ Exacerbations of COPD negatively affect ^[3]^ the condition of the participants’ health and result in a rapidly increasing risk of death, which brings them huge economic and psychological burden.

According to The Global Initiative for Chronic Obstructive Lung Disease (GOLD),^[4]^ the prevention of exacerbations is the major treatment goal for participants with COPD. A large epidemiological study released by the Chinese Center for Disease Control and Prevention in 2018 showed that up to 92.7% of Chinese adult COPD patients are in GOLD stage 1–2,^[5]^ presenting with now very mild or no significant respiratory symptoms, such as movement restriction and dyspnea.^[6]^ For them, the prevention of exacerbations is of great significance.

Traditional Chinese medicine (TCM) has gradually gained worldwide recognition. TCM, which many participants suffered from COPD turn to for help in China, may provide further options for COPD treatment.^[7]^ Recorded in formulary of peaceful benevolent dispensary, Shenling Baizhu Powder has been used in China for more than 900 years, and its clinical efficacy and safety are recognized. Modified Shenling Baizhu Powder comprises 12 herbs including LABLAB SEMEN ALBUM (Bai bian dou), ATRACTYLODIS MACROCEPHALAE RHIZOMA (Bai zhu), PORIA(Fuling), PLATYCODONIS RADIX (Jie geng), NELUMBINIS SEMEN (Lian zi), CODONOPSIS PILOSULA (Dang shen), AMOMI FRUCTUS (Sha ren), DIOSCOREAE RHIZOMA (Shan yao), COICIS SEMEN (Yiyiren), GLYCYRRHIZAE RADIX ET RHIZOMA (Gan cao), Ephedrae Herba (Ma huang), ARMENIACAE SEMEN AMARUM (Ku xing ren).

According to Modern pharmacological studies, Modified Shenling Baizhu Powder has multiple effects such as anti-inflammatory, relieving asthma, antibechic, eliminating phlegm, antioxidant,^[8]^ and the function of immune regulation^[9]^ and improving lung function.^[10]^

In China, Shenling Baizhu Powder is widely used for respiratory diseases. Thus, it is reasonable to hypothesize that Modified Shenling Baizhu Powder is effective for the treatment of COPD. However, few published data show a meaningful effect of Modified Shenling Baizhu Powder on preventing exacerbation and its long-term (18 months) safety profile in participants with COPD of GOLD stage 1 or stage 2. To resolve these issues, we design this randomized, placebo-controlled , double-blind, prospective study protocol.

## 2 Methods/design

### 2.1 Design

**Figure 1:**
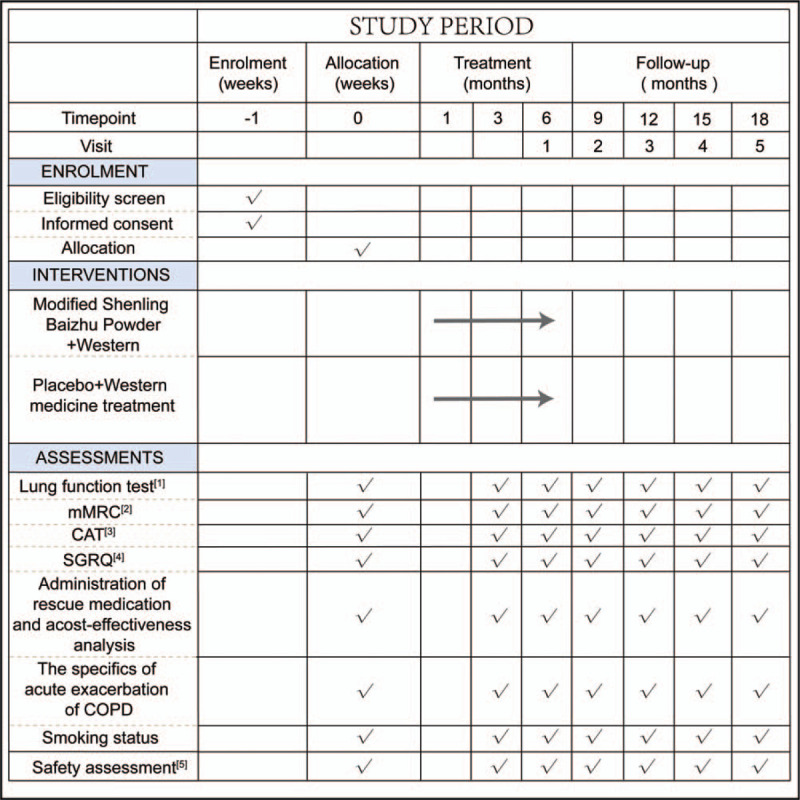
SPIRIT figure: Schedule of enrollments, interventions, and assessments: [1] Lung function tests: forced expiratory volume in 1 second (FEV1), forced volume capacity (FVC), FEV1/FVC; FEV1%pred; [2] mMRC = modified British medical research council; [3] CAT = COPD Assessment Test; [4] SGRQ = St George's Respiratory Questionnaire; [5] safety assessment = blood routine examination, urine routine examination, stool routine examination, liver function test, kidney function test, electrocardiography.

**Figure 2:**
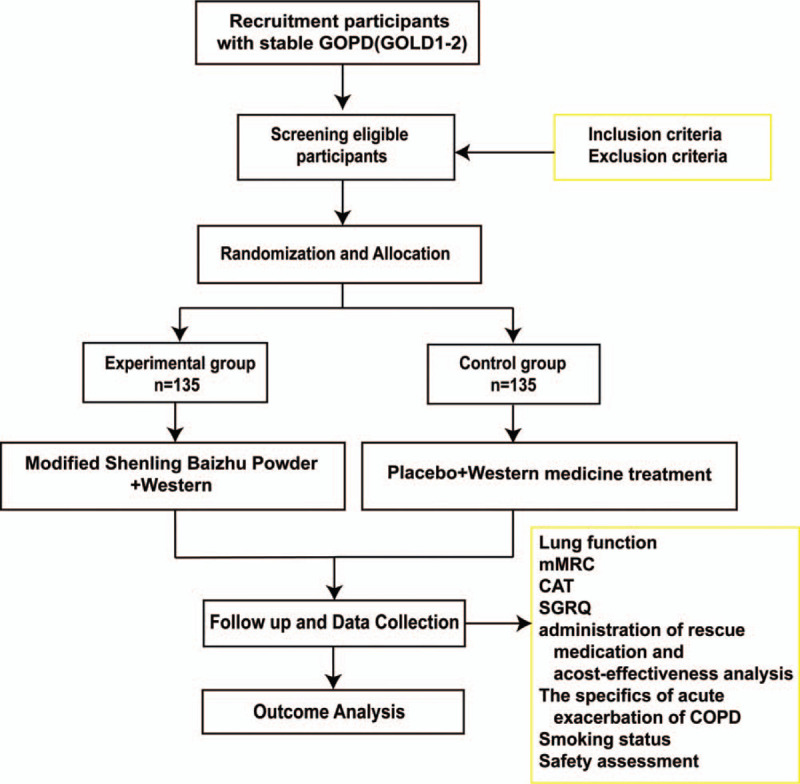
Illustration of the design for clinical studies.

This is a randomized, double-blind, prospective, placebo-controlled trial that will enrol 270 participants with COPD of GOLD stage I or stage II. Participants will be screened after obtaining written informed consent and then randomly assigned to the experimental group or control group in the ratio of 1:1. They will be randomly assigned to receive either 10 g of Modified Shenling Baizhu Powder or a placebo 3 times a day for 6 months while receiving standardized Western medical treatment, and will be followed for 12 months. This study will comply with the Standard Protocol Items: Recommendations for Interventional Trials (SPIRIT) 2013 statement^[11]^ (The SPIRIT figure for registration, intervention, and assessment is shown in Fig. 1; the SPIRIT checklist is in Additional file 1) The flow chart of the study is depicted in Figure 2.

### 2.2 Ethics approval

This study was in accordance with the Declaration of Helsinki (Edinburgh 2000) which has been registered with the Chinese Clinical Trials Registry in 2020 (registration number: ChiCTR2000037873). The study protocol was approved by the China Ethics Review Committee for Registered Clinical Trials (Approval No:ChiECRCT20200227), and its design and implementation were followed up by members of the Ethics Committee. The approval of the Ethics Committee must again be sought for any changes to the protocol.

### 2.3 Recruitment

Participants will be recruited either through the community or through the outpatient department of the Hospital of Chengdu University of TCM (Chengdu, China), where respiratory physicians will screen participants based on inclusion and exclusion criteria. Prior to registration, participants will be informed of details of the clinical study, including its purpose, handling, schedule, and possible risks and benefits, and an informed written consent form must be signed by all participants to be included in the study.

### 2.4 Sample size

The sample size was calculated on the primary outcome (the forced expiratory volume in 1 second (FEV1) at 18 months). According to the previous literature,^[10]^**t**he mean value of FEV1 in the control group is estimated to be 2.54 with a standard deviation of 0.65. After the herbal treatment the mean value of FEV1 in the experimental group is 2.83 with a standard deviation of 0.73. The calculation of the sample size for the experimental and control groups of each was 121 cases using the software Power Analysis and Sample Size version 11.0 (PASS 11.0). Altogether, 268 participants are recruited at a 10% dropout rate. For the actual study, 135 cases per group will be included.

### 2.5 Randomization and allocation concealment

We will use the Central Random System to realize random grouping and random hiding. Subsequent to obtaining written informed consent, 270 random order numbers will be generated by members of the Sichuan TCM evidence-based Medicine Center using SAS 9.2 software (SAS, Cary, NC), and eligible participants will be randomly divided into experimental or control groups at a ratio of 1:1. This process is realized by the network version of the central random system, in which the researchers input the screening information of the expected subjects on the central random system, obtain the subject number, meet the subject standard, and sign the informed consent, and then randomly group and generate the random number, The grouping information of the subjects could not be foreseen by the subject and the researcher, and the random number was systematically managed by Sichuan Evidence-based Medicine Center of Traditional Chinese Medicine. Until the end of the study, the subjects, clinicians, and outcome measures were not aware of the grouping of the subjects.

### 2.6 Blinding

This trial is a double-blind design in which both participants and researchers will not have any awareness of their treatment group during the trial. Herbal and placebo granules are manufactured, packaged, and labeled by Department of Pharmacy, Hospital of Chengdu University of TCM. to assure consistent appearance, shape, odor and specifications. Furthermore, the research team will be instructed not to interact with participants regarding their potential treatment groupings. Researchers should only report to the principal investigator to decide if exposure blinding is appropriate in emergencies, such as serious adverse events, or when the patient requires urgent treatment

### 2.7 Diagnostic criteria

Participants are required to fulfill both the Western medicine diagnostic criteria for COPD (Table 1)^[4]^ and the TCM syndrome diagnostic criteria for lung-spleen qi deficiency syndrome (Table 2).^[12]^ The determination of syndrome differentiation must be made independently by 2 designated TCM associate physicians.

**Table 1:**
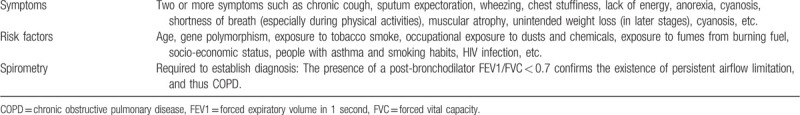
Western medicine diagnostic criteria for chronic obstructive pulmonary disease^[4]^.

**Table 2:**
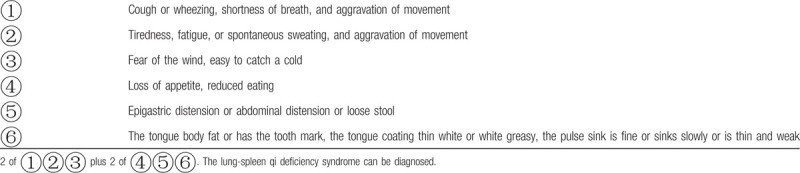
Diagnostic criteria for traditional Chinese medicine differentiation of lung-spleen qi deficiency syndrome^[12]^.

### 2.8 Eligibility criteria

Inclusion criteria:

1. Age range 40–85 years, male or female
2. Grade 1–2 of the COPD diagnostic criteria in GOLD guidelines^[4]^: 20 minutes after inhalation of salbutamol 400, FEV1/FVC <70%, and 50% predicted value ≤FEV1<80% predicted value
3. Participants are in the stable stages of COPD and had no respiratory infections or acute exacerbations of COPD within the past 4 weeks
4. Participants who meet the diagnostic criteria for common TCM syndromes with lung-spleen qi deficiency syndrome
5. Participants volunteer to take part in the study and to provide informed written consent

Exclusion criteria:

1. Patients with chronic lung diseases in which intervention or treatment is required, such as interstitial lung disease, bronchial asthma, active tuberculosis, or bronchiectasis
2. Patients with a combination of severe primary diseases, such as cardiovascular and cerebrovascular diseases, liver and kidney diseases, hematopoietic system diseases, endocrine diseases, severe mental disorders, malignant tumors
3. Patients have evidence of alcohol or drug abuse, known or suspected allergy to the study drug or some of its components, and currently uses oral corticosteroids regularly
4. Women who are breast-feeding, pregnant, or expecting a child
5. Patients who have been involved in any other clinical study in the past 6 months
6. Patients unable to successfully use a dry powder inhaler or perform pulmonary function measurements

### 2.9 Termination and withdrawal criteria

All participants will be notified of their right to stop treatment and withdrawal from the study on any grounds at any time, and the reason for withdrawal will be documented in the case report form (CRF). They will get standardized treatment if they withdraw. The following criteria are used to stop treatment and withdraw from the study project:

1. Patients with severe allergies to the study drug in the course of treatment cannot continue to participate in the clinical study.
2. The participant has poor compliance or is taking other medications prohibited by this study protocol.
3. Loss of follow-up or voluntary withdrawal from the trial.

### 2.10 Test drugs

Test drugs are Modified Shenling Baizhu Powder and Modified Shenling Baizhu Powder mimetic agent (placebo), provided by the Department of Pharmacy, Hospital of Chengdu University of TCM (Sichuan, China). Whole ingredients of Modified Shenling Baizhu Powder are LABLAB SEMEN ALBUM (Bai bian dou)100 g,ATRACTYLODIS MACROCEPHALAE RHIZOMA (Bai zhu100 g, PORIA(Fuling)100 g, PLATYCODONIS RADIX (Jie geng)150 g, NELUMBINIS SEMEN(Lian zi),50 g, CODONOPSIS PILOSULA (Dang shen)100 g, AMOMI FRUCTUS(Sha ren) 50 g, DIOSCOREAE RHIZOMA (Shan yao) 100 g, COICIS SEMEN (Yiyiren) 200 g, GLYCYRRHIZAE RADIX ET RHIZOMA (Gan cao) 100 g, Ephedrae Herba (Ma huang) 50 g, ARMENIACAE SEMEN AMARUM (Ku xing ren) 75 g. One dose every 5 days, 10 g each time, 3 times a day.

The prescription herbs are mixed, processed, filtrated, and pressure spray dried to produce granules, which are packaged into single-dose sachets, each weighing 10 g. Placebos are composed of starch with no active ingredients. By the addition of various food colorings, placebos are made to look and taste as close to real particles as possible.

## 3 Interventions

### 3.1 Treatment plan

Standards Western medicine will be used for both groups. According to GOLD^[4]^ and the Guidelines for the Diagnosis and Treatment of Chronic Obstructive Pulmonary Disease (Revised 2013),^[13]^ participants will receive inhalation treatment with tiotropium bromide powder (Si Li Hua, Boehringer Ingelheim Pharma GmbH & amp; Co. KG [Germany], 18 μg×30 capsules), 18 μg per inhalation, once a day. Also, the proper use of inhalants will be taught to all participants, who would also have to stop smoking and prevent colds. Throughout the study, patients are not allowed to receive other COPD-related medications or herbal treatments except in emergencies.

Experimental group: Participants will take 10 g Modified Shenling Baizhu Powder 3 times daily, dissolved in 100 mL of warm water, for 6 months.

Control group: participants will receive 10 g placebo granules 3 times daily, dissolved in 100 mL of warm water, for 6 months. The measurements will be in accordance with the experimental group.

### 3.2 Outcome measures

Primary outcome: FEV1 after bronchodilator use at 1, 3, 6, 9, 12, 15, 18 months

Secondary outcomes: at 1, 3, 6, 9, 12, 15, 18 months

1. the declines, the between-group difference in the change from baseline to 18 months in FEV1 before bronchodilator use, FVC, FEV1/FVC, FEV1%pred after bronchodilator use;
2. the changes in mMRC score, CAT score, SGRQ score;
3. Frequency, interval, duration, and severity of COPD exacerbations; time to first COPD exacerbation; and the use and cost-benefit analysis of rescue medications; smoking status; safety assessment.

### 3.3 Safety assessment

In China, Shenling Baizhu Powder has a history of over 900 years, and the dose used in this trail is within the recommended range according to the People's Republic of China Pharmacopeia (2015 edition). In addition, we will assess the safety of Modified Shenling Baizhu Powder using a range of measures, including subjective descriptions and laboratory tests, with particular attention to gastrointestinal intolerance and cardiac, hepatic, and renal impairment, from enrollment through follow-up.

### 3.4 Compliance

Upon randomization of participants, investigators at the study site will use all reasonable efforts to follow up with participants throughout the study. Adherence to the intervention will be monitored on each visit, and participants will be required to have all study containers returned with any unused granule packets, including all empty containers. Messages will be sent prior to each visit via WeChat or phone to remind participants of the impending data collection. Furthermore, ongoing support will be offered to participants during the follow-up phase, including free registration and treatment counselling.

### 3.5 Adverse events

Any adverse events will be recorded in case report files (CRF) irrespective of their relationship to the study intervention. The intervention will be stopped immediately in the event of any serious adverse event. The timing of the intervention, severity, relationship to the drug, and actions taken in accordance with the China Food and Drug Administration (CFDA) Standard Operating Procedures (SOPs) will be documented in detail. Furthermore, severe adverse events shall be reported to the Steering Committee and Ethics Committee in 24 hours.

### 3.6 Data management and quality control

All records will be collected in the CRFS and filled out by a trained and qualified investigator. The original record will not be altered once the CRF is completed, even if any modifications are made. The clinical inspector will review the completed CRFs. Data entry and management will be directed by a medical statistics specialist. To ensure data accuracy, 2 data managers will independently enter and proofread data. Once the database created is reviewed and confirmed to be correct, the data will be locked by the principal investigator and statistical analysts. The locked data or files will not be altered thereafter and will be presented to the research team for statistical analysis. Data monitoring will be the responsibility of The Sichuan TCM evidence-based Medicine Center (Chengdu, China), which has no competing interests. Data will be reviewed in the middle of the trial by The Department of Science Research of the Hospital of Chengdu University of TCM which is independent of the investigators.

### 3.7 Statistical analysis

The intention to treat principle will be followed for all data analysis. The Statistical Package for the Social Sciences version 22.0 (SPSS 22.0, Chicago, IL) will be used to analyze the data. The analysis method should be based on the characteristics of the data distribution: The measurement data will be expressed as mean ± standard deviation and normal distribution, paired *t* test will be used for comparison before and after treatment, *t* test will be used for between-group comparisons (homogeneity test of variance will be performed with 0.05 as the test standard), and rank sum test will be used for comparison between groups. Chi-square test will be used for enumeration data, and rank-sum test will be used for grading data. Bilateral tests will be used for all tests, with *P* < .05 indicating a significant difference. For baseline data, the full analysis set (FAS, including completed cases and dropout cases, excluding censored cases) is used, and the full analysis set and the per-protocol set(PP set, including completed cases, excluding dropout cases and censored cases) are used for efficacy evaluation, which can enhance the reliability of trial results when the conclusions of the two analyses are consistent. When inconsistencies arise, differences should be fully discussed and explained. In the analysis of the full analysis set, the missing data are substituted by the method of sequence mean value.

## 4 Discussion

In China, COPD is a common chronic respiratory disease, and the present situation on the prevention and control of this chronic disease is not optimistic. Characterized by high morbidity, mortality, and disease burden, COPD has emerged as one of the most salient public and medical problems.^[14]^ Although modern medicine has been very effective in alleviating the symptoms of COPD, these measures cannot fully control disease progression and prevent acute exacerbations. New interventions are still needed to improve efficacy.^[15]^ TCM has long been extensively used in the treatment of COPD in China due to its low probability of adverse effects and low cost.^[16]^ With a history of use of several hundred years, Modified Shenling Baizhu Powder is a commonly used formula for the treatment of stable COPD.

But to date, the efficacy and safety of Modified Shenling Baizhu Powder in early-stage COPD has rarely been uniformly understood. Therefore, a prospective and convincing trial is necessary to demonstrate the risks and benefits of adding Modified Shenling Baizhu Powder to the therapy of participants with COPD based on syndrome differentiation. As far as we know, it is the first clinical trial to investigate the effectiveness of Modified Shenling Baizhu Powder with Western medicine in the delaying of early-stage COPD (GOLD stage I–II) lung function deterioration. We will focus on lung function, CTA, mMRC, SGRQ, quality of life, and safety in both treatment and follow-up phases. In our trail, we will use effective objective tools like lung function tests, which improve the reliability and generalizability of the results.

The study has some limitations. First, since the study was conducted in Sichuan, China, the relative effects of the test drugs are unclear whether they would be comparable among other ethnic groups. Second, in clinical practice, participants with pure COPD are rare and most of them are associated with other diseases such as hypertension, diabetes, and coronary heart disease. Therefore, the utility (extrapolation) of the results of this study is unclear and requires further evaluation. That said, the results shall aid the decision-making process for COPD therapy and management as well as provide important information which could be incorporated into guidelines for future treatment. A multicenter randomized controlled trial should be conducted in the future, to investigate a large sample of COPD participants and implement a multidimensional comprehensive evaluation.

## Acknowledgment

The authors are grateful to Dr Chuantao Zhang for his assistance and valuable advice.

## Author contributions

**Conceptualization:** Xiaohong Xie, Keling Chen.

**Investigation:** Jianqin Chen, Jurong Zeng.

**Supervision:** Wujun Wang, Wei Xiao.

**Writing – original draft:** Yufei Liu, Keni Zhao.

**Writing – review & editing:** Jing Xiao.
